# Advancing intraoperative tumour detection and molecular-guided precision surgery, FDA’s approval of pafolacianine injection: an editorial

**DOI:** 10.1097/MS9.0000000000001534

**Published:** 2023-11-20

**Authors:** Mahnoor Fayaz, Khabab Abbasher Hussien Mohamed Ahmed

**Affiliations:** a Department of Internal Medicine, Ayub Medical College, Abbottabad, Pakistan; b Faculty of Medicine, University of Khartoum, Khartoum, Sudan

*Dear Editor,*

Ovarian cancer, one of the most fatal cancers in women worldwide, is typically diagnosed in its later stages when it has already disseminated to the peritoneal cavity. Complete surgical excision, which is the standard therapy and highly necessary at later stages, becomes difficult, and the probability of any remaining tumour tissue, even after surgery, is still very high. The presence of any residual tumour nodules can result in poor prognosis and recurrence and is also one of the reasons for the low 5-year survival rate of patients at stages III and Ⅳ of cancer advancement. Since excision of all tumour margins is hard, better tumour identification strategies and high-resolution intraoperative imaging are needed^1,2^. Over the years, various techniques have been sanctioned for intraoperative visualization of tumours, including magnetic resonance imaging (MRI), ultrasonography, and positron emission tomography (PET)^2^.

Apart from these, surgeons use palpation and inspection for the identification of tumour lesions; these techniques are not reliable and pose a high chance of any occult lesion remaining obscured^3^. To tackle this issue effectively, a highly unique and effective strategy has been explored, including fluorescent agents such as pafolacianine for accurate visualization of malignant lesions^4^. We write this correspondence intending to provide information regarding new emerging ways of accurate diagnostic and prognostic tools regarding malignancies, U.S. Food and Drug Administration (FDA)-approved intraoperative imaging agents, and recommendations to efficiently use them for improved treatment of ovarian malignancies in the future.

Many new agents have been developed for better therapeutic approaches, including antibody–dye conjugates, multifunctional nanoparticles, and small-molecule fluorophores. Owing to the large size of antibody-conjugated dyes, they accumulate in various excretory tissues and are usually unable to penetrate deep into the tumour mass. However, multifunctional nanoparticles tend to be unsafe owing to various biodistribution-related issues. Hence another approach has been brought into use after FDA approved the use of indocyanine dye (ICG), which is highly effective in the visualization of tumours during debulking surgeries but due to various limitations including specificity, sensitivity, and poor tumour-to-background ratio (TBR), a need for a better agent is highly felt^1^. Due to these and various other diagnostic and prognostic limitations, ovarian cancers have been highly fatal. Better surgical approaches, improved imaging technologies, and accurate malignant tissue visualization are therefore desired more than ever now.

Pafolacianine injection, an agent approved by the FDA^5^, has shown promising results and is, therefore, a potential intraoperative imaging agent. This fluorescent imaging agent is comprised of a folate analogue conjugated with an NIR (near-infrared) cyanine dye, S0456. Also known as OTL38, pafolacianine absorbs light in the near-infrared spectrum and particularly binds to folate receptor alpha (FRα). FRα is highly upscaled in many malignant tissues of breast, ovarian, cervical, lung, brain, and kidney origins^4^. The fact that pafolacianine binds specifically to FRα receptors present in tumour tissues and not the normal ones deems it to be highly effective for contrast imaging as its negative impacts on normal tissues would be the fewest.

We label a contrast agent ‘ideal’ when it can explicitly enter the malignant tissues, possess fewer toxic effects for the otherwise normal tissues, and can be viewed at pronounced depths below the target tissue. Pafolacianine is a highly potent imaging agent and is said to let the intraoperative imaging take within 2 h of infusion, can be synthesized quite readily, stays stable for longer periods, and allows precise identification of occult tumour nodules and excision of tumour margins that otherwise can lead to the recurrence of malignancies^2^. A randomized phase 3 study of pafolcianine injection showed how various adverse effects consistent with the use of pafolacianine injection, including nausea and vomiting and transient infusion reactions, were manageable. This study also recommended the use of anti-emetics preceding treatment by pafolacianine to cope with such adverse effects^6^. Another phase 2 clinical trial, which included 29 patients with ovarian cancer who received pafolacianine injection ahead of surgery, showed a positive predictive value of 94.93%. Eight out of 29 patients went through a few mild adverse effects including nausea, vomiting, infusion reactions and abdominal pain^7^.

Apart from pafolacianine, many other fluorescent dyes have been developed; however, owing to various limitations, they are deemed unsuitable for the accurate identification of tumour tissues. Dyes like 5-aminolevulinic acid and EC17, a folate-targeted derivative of fluorescein and ICG, were developed. While 5-aminolevulinic acid is unable to penetrate solid tissues, EC17, due to its emission in the visible wavelength range, is unable to image buried tumour masses^2^. Phase 3 clinical trials have shown that this novel imaging agent can be a beacon of hope in the treatment of ovarian cancer^6^. This highly devastating epithelial ovarian cancer, often termed as ‘silent lady killer’ is the fifth biggest cause of death among women^3^. This calls for an ever-increasing need for devising ways and therapeutic regimens that are more accurate and reliable.

Pafolacianine can be a very promising tool in the realm of precision surgery. We highly recommend the use of pafolacianine sodium injections in surgical and oncological fields. Large-scale clinical trials and research are required to standardize procedures and investigate the long-term use of such contrast agents on patients. This advancement in precision surgery should be highly utilized, and such adjunct agents should be made available during surgical procedures to enhance patient survival. Despite substantial evidence of the prognostic efficacy of pafolacianine via multiple studies, we highly recommend the persistence of animal studies of this agent to report adverse effects, various preliminaries before its use, relative effectiveness, and diagnostic and prognostic performance. More comprehensive studies on such novel and potential intraoperative imaging agents are a contemporary requirement that should not be overlooked at any cost.

## Ethical approval

Ethics approval was not required for this Editorial article.

## Consent

Informed consent was not required for this Editorial article.

## Sources of funding

None.

## Author contribution

M.F.: conceptualization, data curation, writing the original draft, review and editing; K.A.H.M.A.: supervision, reviewing and editing.

## Conflicts of interest disclosure

There are no conflicts of interest.

## Research registration unique identifying number (UIN)

1. Name of the registry: not required.
2. Unique identifying number or registration ID: not applicable.
3. Hyperlink to your specific registration (must be publicly accessible and will be checked): not applicable.

## Guarantor

Dr Khabab Abbasher Hussien Mohamed Ahmed, Faculty of Medicine, University of Khartoum, Khartoum 11111, Sudan; e-mail: Khabab9722@gmail.com, ORCID: https://orcid.org/0000-0003-4608-5321

## Data availability statement

The data that supports the findings of this study is available with the corresponding author upon reasonable request.

## Provenance and peer review

Not commissioned, externally peer-reviewed.
